# Fate, transport, and biological impacts of microplastics in agricultural soil-wheat systems: from soil movement to food safety

**DOI:** 10.3389/fpls.2026.1906511

**Published:** 2026-08-03

**Authors:** Umair Sarfraz, Quan Ma, Min Zhu, Jinfeng Ding, Chunyan Li, Wenshan Guo, Xinkai Zhu

**Affiliations:** 1 Jiangsu Key Laboratory of Crop Genetics and Physiology/Jiangsu Key Laboratory of Crop Cultivation and Physiology, Agricultural College of Yangzhou University, Yangzhou, Jiangsu, China; 2 Co-Innovation Center for Modern Production Technology of Grain Crops, Yangzhou University, Yangzhou, Jiangsu, China; 3 Joint International Research Laboratory of Agriculture and Agri-Product Safety, The Ministry of Education of China, Yangzhou University, Yangzhou, Jiangsu, China

**Keywords:** agricultural soil, food safety, grain quality, microplastics, plastisphere, root uptake, soil transport, wheat

## Abstract

Microplastics are persistent contaminants of agricultural soils and may affect soil structure, microbial activity, nutrient cycling, crop growth, and the quality of plant-derived foods. Wheat (*Triticum aestivum* L.) deserves specific attention because it is a major staple crop, develops an extensive fibrous root system in cultivated soil, and produces grain that is consumed directly by humans. These characteristics create a close connection among soil contamination, rhizosphere processes, crop performance, and possible food-chain exposure. Unlike broader reviews of microplastics in crops, this review follows the pathway from particle entry into wheat-growing soils to transport, transformation, rhizosphere interactions, wheat responses, and possible contamination of edible grain. The movement and effects of microplastics depend on particle size, shape, polymer type, density, surface properties, aging, soil texture, mineral composition, organic matter, water movement, root activity, and soil organisms. Available studies show that plastic particles can alter root development, nutrient acquisition, oxidative balance, photosynthesis, biomass, and yield, although the direction and magnitude of these effects differ among experimental conditions. Evidence for root internalization and vascular transport is strongest for nanoplastics and submicrometer particles under controlled conditions. Direct field evidence for the accumulation of larger microplastics in mature wheat grain remains limited. Important research gaps include the scarcity of field measurements, the use of unrealistic exposure concentrations, poor separation of microplastic and nanoplastic evidence, uncertain root-to-grain transfer, and the lack of standardized analytical methods. Future research should combine realistic field exposure, aged and mixed-polymer particles, multi-season experiments, advanced particle tracing, and contamination-controlled analysis of wheat tissues and grain.

## 1 Introduction

Microplastic pollution is an emerging concern in terrestrial ecosystems and is widely detected in agricultural soils (Rillig, 2012; de Souza MaChado et al., 2018a; Piehl et al., 2018). Microplastics are defined as plastic particles that are smaller than 5 mm, originating from the breakdown of larger plastics as well as from direct inputs like mulching films, sewage sludge, compost, irrigation water, polymer-coated fertilizers, and deposition of atmospheric dust to the soil (Duis and Coors, 2016; Nizzetto et al., 2016; Bläsing and Amelung, 2018). Their behavior in soil after introduction is a function of the type of polymer, the particle size and shape, density, chemistry, age, and interactions with soil biota and organic matter (Duis and Coors, 2016; Rillig et al., 2017a; Bläsing and Amelung, 2018). The slow removal of microplastics from agricultural soils, combined with regular inputs from farming activities, means that soils are long-term sinks for microplastics and that the continual presence of microplastics may affect soil physical functions and crop performance (Bläsing and Amelung, 2018; de Souza MaChado et al., 2019).

Previous reviews have summarized the direct effects of soil microplastics on seed germination, root development, plant physiology, and yield, as well as indirect effects stemming from changes in soil properties, microorganisms, nutrient availability, and interactions with other contaminants (Iqbal et al., 2023). However, findings from different crops, particle-size classes, growth media, and exposure conditions have often been considered together. As a result, it is difficult to determine which conclusions are directly supported for wheat and which are based on vegetables, other cereals, hydroponic systems, or experiments involving mainly nanoplastics. The present review addresses this limitation by examining the complete pathway from microplastic entry into wheat-growing soils to soil transport, rhizosphere exposure, wheat responses, and possible food-safety consequences. It also separates evidence for microplastics from evidence for nanoplastics and distinguishes observed processes from proposed pathways. A wheat-specific assessment is needed because of the crop’s root characteristics, exposure conditions, dietary importance, and the limited extent to which findings from other plant systems can be transferred directly to wheat. Wheat forms a dense fibrous root system that occupies a large volume of cultivated soil and creates extensive contact among roots, soil pores, microorganisms, organic matter, minerals, and plastic particles. Root elongation, water uptake, rhizodeposition, and the formation of root channels may influence particle retention and redistribution in the rhizosphere. At the same time, the accumulation of particles around roots may alter water and nutrient acquisition and subsequently affect wheat physiology, yield formation, and grain quality. Because wheat grain is widely consumed, it is also necessary to determine whether plastic particles can reach edible tissues during plant growth or whether reported contamination is mainly related to foliar deposition, harvesting, storage, and processing. Wheat therefore provides a useful soil-crop-food system in which environmental transport, rhizosphere processes, plant responses, and possible human exposure can be considered together.

Recent studies illustrate why particle size, polymer type, soil conditions, and experimental design must be considered separately. Tumwet et al. (2024) showed that polyester fibers and PVC fragments followed different vertical movement patterns in sandy soil containing wheat, with fibers strongly attaching to roots and PVC fragments moving more freely through the soil profile. Zhuang et al. (2024) compared 50-nm polyethylene nanoplastics with 10-μm polyethylene and polystyrene microplastics and found that wheat and rhizosphere responses varied with particle size, polymer type, and concentration. Yang et al. (2025) further showed that kaolinite and montmorillonite reduced the accumulation of 200-nm polystyrene nanoplastics in wheat roots and shoots. Under more environmentally representative soil exposure, Ziajahromi et al. (2026) found that plant presence reduced the leaching of microplastics and that aged, but not pristine, nanoplastics were internalized by wheat. Taken together, these studies show that findings from one particle class or exposure system should not be generalized to all microplastics in wheat production. Under realistic soil conditions, plant presence reduced the downward leaching of microplastics. In the same experiment, smaller particles showed greater downward mobility than larger particles (Ziajahromi et al., 2026). These were separate findings, and the greater mobility of smaller particles should not be presented as the cause of plant-mediated particle retention.

Microplastics may alter soil aggregation, porosity, water retention, pH, organic-carbon distribution, microbial-community structure, enzyme activity, and nutrient cycling. These responses are not consistent across studies and depend on polymer type, particle size, shape, concentration, aging, exposure duration, soil texture, moisture, and organic matter (Khalid et al., 2020; Wang et al., 2022; Aralappanavar et al., 2024). Evidence from combined-contaminant experiments should be interpreted separately from evidence obtained with microplastics alone. For example, changes observed during the combined exposure of wheat to microplastics and metals cannot be attributed entirely to the plastic particles because the metal and its interaction with the particles may contribute to the response. Such studies are still relevant because microplastics can alter the mobility or bioavailability of associated contaminants, but the individual and combined effects must be clearly distinguished.

Several unresolved questions currently prevent a reliable assessment of microplastic risks in wheat production. First, the concentrations, size distributions, polymers, and aging states of particles in wheat-growing fields have not been adequately quantified. Second, many experiments use pristine particles, single polymers, short exposure periods, or concentrations that may not represent agricultural soils. Third, evidence for root internalization and vascular transport is derived mainly from nanoplastics or submicrometer particles studied under controlled conditions, making its relevance to larger microplastics in field soil uncertain. Fourth, direct evidence distinguishing root-to-grain transport from foliar deposition and post-harvest contamination remains scarce. Fifth, soil minerals, aggregation, organic matter, and rhizosphere processes are not consistently included in plant-uptake assessments. Finally, differences in sampling, digestion, identification, and quantification methods make comparisons among studies difficult. These gaps must be addressed before reliable conclusions can be drawn about crop productivity, grain contamination, and food-safety risk.

### 1.1 Objectives

This review uses a source-to-receptor framework that links agricultural plastic inputs with soil transport and transformation, rhizosphere exposure, wheat responses, and possible food-chain outcomes. It addresses four main questions:

1. Which particle properties, soil conditions, biological processes, and agricultural practices control the retention and redistribution of microplastics in wheat-growing soils?
2. How do changes in soil structure, microorganisms, enzyme activity, and nutrient cycling influence wheat growth, physiology, yield, and grain quality?
3. What evidence supports particle attachment, root internalization, root-to-shoot transport, and possible root-to-grain transfer, and how does this evidence differ between microplastics and nanoplastics?
4. Which methodological limitations and field-data gaps prevent reliable assessment of agronomic and food-safety risks?

The review compares areas of agreement and disagreement among studies, separates demonstrated mechanisms from proposed pathways, and identifies priorities for field-relevant research.

## 2 Sources, types, and characteristics of microplastics in agricultural soils

Agricultural soils receive microplastics from several recurring sources, including plastic mulch residues, organic amendments, irrigation water, coated fertilizers, and atmospheric deposition. Plastic mulch and films are commonly used for soil moisture, weed control, temperature control, and increased yields (Zubris and Richards, 2005; Kasirajan and Ngouajio, 2012; Steinmetz et al., 2016; Weithmann et al., 2018; Corradini et al., 2019; Ragoobur et al., 2021; Diao et al., 2023). These films break down into microplastics under repeated mechanical stress, such as tillage, exposure to UV radiation, and weathering. Research has demonstrated that broken PE and PP residues can persist in soil for several crop seasons, altering soil porosity and water infiltration rates (Dris et al., 2016; Bandopadhyay et al., 2018; Cao et al., 2020; Hu et al., 2020; Li et al., 2022).

Another significant source is sewage sludge and biosolids, which carry microplastics from wastewater and deposit them in croplands (Zubris and Richards, 2005; Corradini et al., 2019; Crossman et al., 2020). Treated sludge often contains textile fibers (polyester and polyamide), as well as PE, PP, PVC, and PET fragments. Sludge has the potential to cause long-term changes in soil microbial community composition and nutrient cycles through repeated applications (Awet et al., 2018; Boots et al., 2019). Some research indicates that microplastics in sludge may bind to heavy metals and organic pollutants, thereby affecting their bioavailability to crops (Hodson et al., 2017; Guo and Wang, 2019).

Microplastic contamination of compost and digestate comes from contaminated municipal biowaste, packaging, and residues (Weithmann et al., 2018; Tian et al., 2022; Steiner et al., 2023). Although composting can alter particle surfaces by heat, microbial colonization, and oxidation, it does not completely degrade the microplastic particles, and their presence will remain. These particles can interact with soil aggregates and alter rhizosphere processes, thereby affecting nutrient uptake by wheat (Lehmann et al., 2017; de Souza MaChado et al., 2018b; Tayyab et al., 2024).

Microplastic particles also enter agricultural soil when irrigation water, particularly reclaimed wastewater or surface water contaminated with microplastics, is applied (Li et al., 2020; Ragoobur et al., 2021). Depending on particle size, density, and water movement, these microplastics can either remain on the soil surface or sink into the root zone. The evidence suggests that smaller microplastics (<300 µm) are more mobile and can be transported deeper into the soil than the larger pieces (O’Connor et al., 2019; Yu et al., 2019).

Microplastic contamination can occur when polymer-coated fertilizers are applied as fertilizer (Diao et al., 2023; Bhattacharjee et al., 2025). These coatings are meant to be slowly released over time, but may remain in soil as microplastic particles once nutrients have been dissolved. High concentration microplastic hotspots may occur in specific regions around the application of fertilizers, which may affect the composition of the microbial communities in the rhizosphere and the access to available nutrients (Fei et al., 2020; Zhao et al., 2022).

Microplastics can be deposited in the atmosphere as a continuous source, especially in the form of fibers and dust particles from urban, industrial, and traffic-related emissions (Dris et al., 2016; Cai et al., 2017). Soil surfaces, plant leaves, and stems may be targets for deposition, which can then be washed into the soil via irrigation or rainfall. It has been reported that the sources of the atmospheric fibers are emissions from textiles, tire wear, and construction dust, and that they can interact with soil aggregates, thus affecting water retention and microbial activities (Kole et al., 2017; Zhang et al., 2019; Gavigan et al., 2020). Major sources, dominant polymer types, and relevance of microplastics in agricultural soil-wheat systems are described in **Table 1**.

**Table 1:** Major sources, dominant polymer types, and relevance of microplastics in agricultural soil-wheat systems.

Source	Typical particle forms	Common polymers	Main entry pathway	References
Plastic mulch and films	Films, fragments, sheets	PE, PP, biodegradable	Weathering, tearing, tillage	(Kasirajan and Ngouajio, 2012; Steinmetz et al., 2016; Cao et al., 2020; Hu et al., 2020; Li et al., 2022).
Sewage sludge/biosolids	Fibers, fragments, films	Polyester, PA, PE, PP, PET, PVC	Land application	(Zubris and Richards, 2005; Hodson et al., 2017; Awet et al., 2018; Boots et al., 2019; Corradini et al., 2019; Guo and Wang, 2019; Crossman et al., 2020).
Compost/digestate	Fragments, films, fibers	PE, PP, PET, PS, biodegradable	Organic amendment application	(Lehmann et al., 2017; de Souza MaChado et al., 2018b; Weithmann et al., 2018; Tian et al., 2022; Steiner et al., 2023; Tayyab et al., 2024).
Irrigation water	Small fragments, fibers, particles	Mixed polymers	Wastewater reuse or surface water	(O’Connor et al., 2019; Yu et al., 2019; Li et al., 2020; Ragoobur et al., 2021).
Polymer-coated fertilizers	Coating fragments, capsules	PE, PU, epoxy resins	Fertilizer application	(Fei et al., 2020; Zhao et al., 2022; Diao et al., 2023; Bhattacharjee et al., 2025).
Atmospheric deposition	Fibers, dust, light fragments	Polyester, PA, PE, PP, tire-wear particles	Dry and wet deposition	(Dris et al., 2016; Cai et al., 2017; Kole et al., 2017; Zhang et al., 2019; Gavigan et al., 2020).

Field surveys show considerable variation in microplastic concentrations among agricultural soils. In the United Kingdom, Cusworth et al. (2024) reported 1,320–8,190 particles kg^−1^ in soils used for carrot and potato production, with a mean concentration of approximately 3,680 particles kg^−1^. Fields with a recent history of plastic crop-cover use contained more particles than fields where crop covers had not been used. In agricultural soils from Hainan Island, China, Khan et al. (2023) reported 2,800–82,500 particles kg^−1^, with a mean of 15,461.52 particles kg^−1^. Particles between 20 and 200 μm were the most abundant size class, fragments were the dominant shape, and polyethylene and polypropylene were the main polymers. These studies were not limited to wheat fields, but they show the range of contamination that can occur in intensively managed agricultural soils. Direct comparisons should be made cautiously because reported concentrations depend on sampling depth, minimum detectable particle size, extraction and digestion procedures, polymer-identification methods, and reporting units. The environmental fate of microplastics in soil associated with agriculture is influenced by the type of polymer, size, shape, density, surface chemistry, and aging state of the microplastic (Duis and Coors, 2016; Nizzetto et al., 2016; Bläsing and Amelung, 2018; Rillig et al., 2019). Fibers become entangled in soil and roots, fragments can be lodged between soil particles, and films can create water restrictions. Bioplastics can break down more quickly under favorable conditions but may still contain microplastic residues that can persist for several cropping cycles (Sintim and Flury, 2017; Bandopadhyay et al., 2018). Understanding these sources and characteristics is critical for assessing the fate and impacts of microplastics in wheat-based systems.

**Figure 1:**
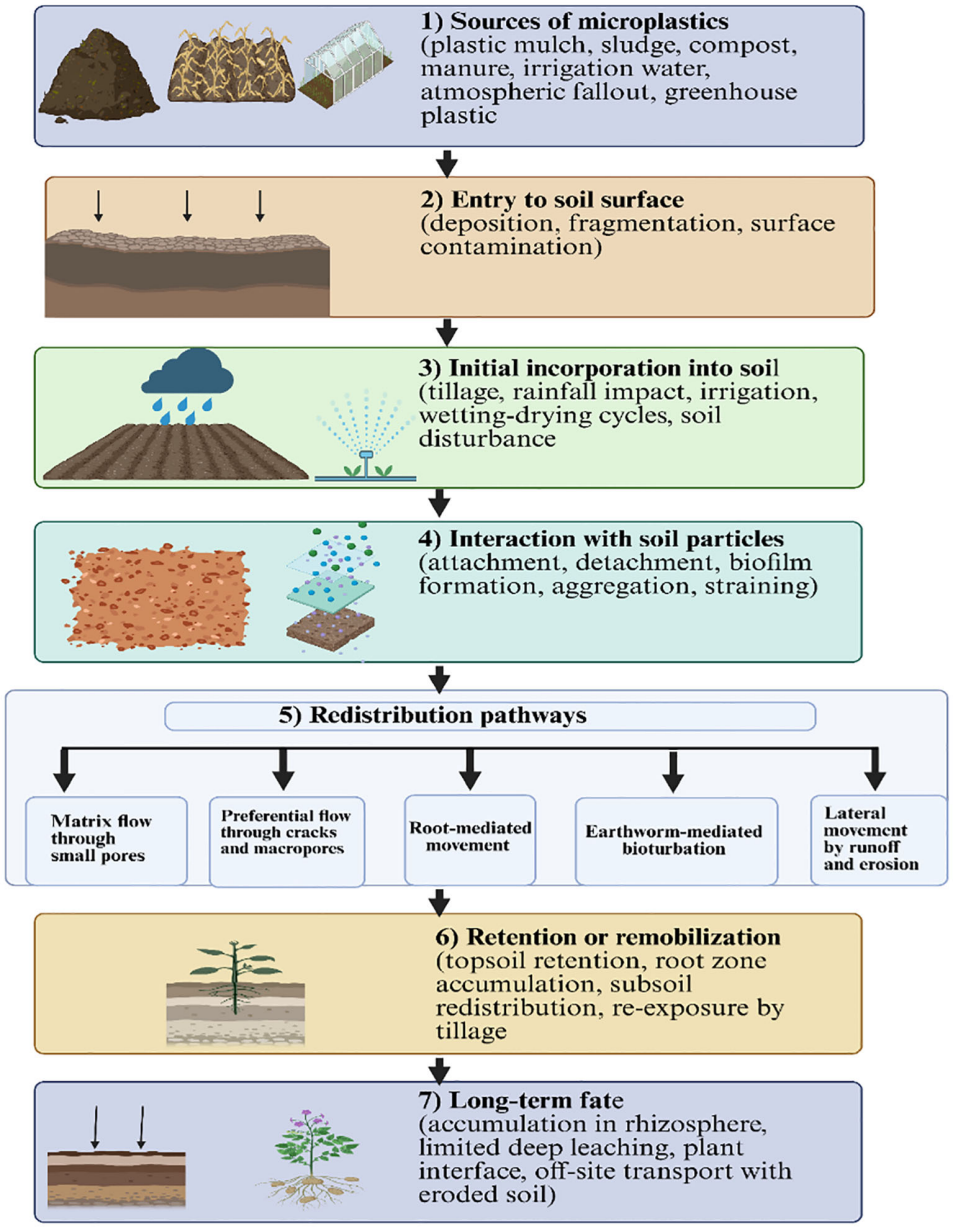
Conceptual sequence of microplastic entry, incorporation, interaction, redistribution, and long-term fate in agricultural soils. Microplastics enter agricultural soils through plastic mulch residues, sewage sludge and biosolids, compost and organic amendments, manure, irrigation water, atmospheric deposition, and greenhouse-related plastic materials. Following deposition at the soil surface, particles may be incorporated into the soil through tillage, rainfall, irrigation, wetting-drying cycles, and other disturbances. Interactions with soil particles, minerals, organic matter, aggregates, and microbial biofilms influence subsequent matrix flow, preferential transport through cracks and macropores, root-mediated redistribution, fauna-assisted bioturbation, runoff and erosion, retention, and remobilization. The relative importance of these pathways depends on particle properties, soil conditions, hydrology, biological activity, and agricultural management.

The occurrence of microplastics in agricultural soil reflects both the quantity of plastic entering the system and the processes that redistribute or retain particles after entry. Agricultural practices, wastewater-derived materials, atmospheric deposition, surface runoff, land use, particle characteristics, soil properties, erosion, and microbial activity may all influence the resulting distribution (Zheng et al., 2025). These factors should be considered when comparing microplastic concentrations among wheat-growing regions because similar inputs may produce different soil distributions under different environmental and management conditions. The overall sequence from agricultural microplastic inputs to soil incorporation, particle–soil interactions, redistribution, retention, and long-term fate is summarized in **Figure 1**.

## 3 Movement and transport of microplastics in agricultural soils

**Figure 2:**
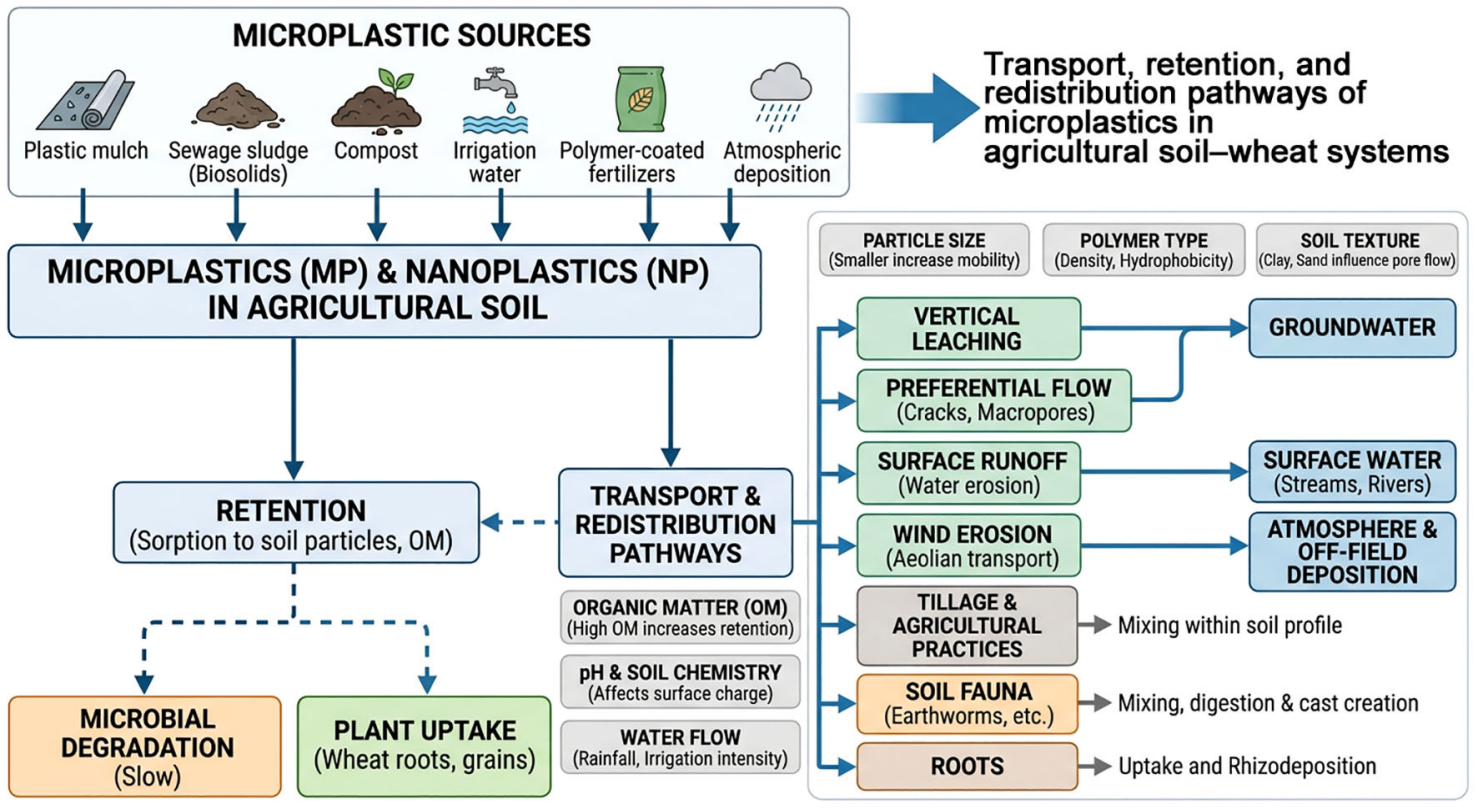
Transport, retention, and redistribution pathways of microplastics and nanoplastics in agricultural soil-wheat systems. Plastic particles enter agricultural soils through mulch residues, sewage sludge and biosolids, compost, irrigation water, polymer-coated fertilizers, and atmospheric deposition. Following entry, particles may be retained through attachment to soil particles and organic matter or redistributed through vertical leaching, preferential flow through cracks and macropores, surface runoff, wind erosion, tillage, root activity, and soil-fauna processes. Particle mobility and retention are controlled by particle size, polymer type, density, surface properties, soil texture, organic matter, pH, soil-solution chemistry, and water flow. Solid arrows indicate principal or comparatively well-supported pathways, whereas dashed arrows indicate context-dependent, indirect, or less certain processes. MP, microplastic; NP, nanoplastic; OM, organic matter.

The transport of microplastics in agricultural soils is controlled by interactions among particle properties, soil characteristics, hydrological conditions, biological activity, and agricultural management. Relevant particle properties include size, density, shape, polymer composition, surface charge, aging, and biofilm development. Soil controls include texture, pore-size distribution, aggregate stability, mineral composition, organic matter, pH, and soil-solution chemistry. Rainfall, irrigation, infiltration, wetting–drying cycles, and surface runoff provide hydrological driving forces, whereas root growth, soil fauna, tillage, and machinery create or modify transport pathways. These factors act together and may either increase particle mobility or promote retention and aggregation. Microplastic distribution is therefore expected to vary among the soil surface, plow layer, rhizosphere, preferential-flow channels, and deeper soil horizons (Rillig et al., 2019; Park et al., 2023; Zheng et al., 2025). The principal transport, retention, and redistribution pathways and their major controlling factors are summarized in **Figure 2**.

### 3.1 Vertical transport

There are several mechanisms for the vertical movement of microplastics, including matrix flow, preferential flow, and bioturbation. Particle size, density, shape, and surface chemistry play a significant role in the extent and rate of microplastic downward transport (Huerta Lwanga et al., 2016; Sintim and Flury, 2017; Zhang and Liu, 2018; Rillig et al., 2019; Park et al., 2023). Smaller particles (<300 µm) and nanoplastics can enter soil pores and accumulate in subsoil layers over time, whereas larger particles and fibers are mostly held in the plough soil or in aggregates (Sintim and Flury, 2017; Zhang and Liu, 2018). Even larger particles may travel deep in the root zone through preferential flow around cracks, macropores, and root channels (Huerta Lwanga et al., 2016; Li et al., 2020a; Zhao et al., 2022). Small fragments may move deeper during repeated wetting–drying cycles, whereas fibers may be retained around roots or within soil aggregates (Laermanns et al., 2021; Schefer et al., 2025; Severe et al., 2025; Soltani Tehrani et al., 2025). Burrowing by earthworms or casting by birds and animals can create additional pathways, thereby increasing the likelihood of heterogeneous particle redistribution throughout the soil profile (Rillig et al., 2017b; Rillig and Lehmann, 2020). Vertical movement may be retarded or accelerated by interactions with soil organic matter, humic substances, and mineral surfaces. Clay minerals, iron and aluminum oxides, and other charged soil components may promote particle attachment, electrostatic repulsion, cation bridging, or heteroaggregation. The direction of these interactions depends on particle surface chemistry, mineral charge, pH, ionic strength, and dissolved organic matter. Aggregation may increase effective particle size and promote retention or pore blockage, whereas changes in soil-solution chemistry may release previously retained particles. Yang et al. (2025) found that kaolinite and montmorillonite formed heteroaggregates with 200-nm polystyrene nanoplastics and reduced their accumulation in wheat roots and shoots. Because this experiment concerned nanoplastics, the results should not be applied directly to larger microplastic fibers or fragments.

### 3.2 Horizontal transport

Horizontal transport occurs through surface runoff, wind erosion, tillage, and irrigation. Rainfall-induced runoff can mobilize surface microplastics downslope, with transport affected by rainfall characteristics, vegetation cover, soil roughness, and particle properties (Han et al., 2022; Severe et al., 2025). Light fibers and dust-like particles may be resuspended by wind and redistributed within or between fields (Rillig et al., 2017b; Wan et al., 2019). Microplastics are vertically and horizontally distributed as a result of tillage, during which tillage equipment helps mix them into the plough layer (Li et al., 2020a; Schefer et al., 2025). Irrigation may facilitate lateral movement along the soil surface or through shallow subsurface pathways, with transport depending on particle size, water velocity, soil moisture, surface slope, and soil structure (Li et al., 2020a; Laermanns et al., 2021). Transport pathways of microplastics in agricultural soils, along with key controlling factors, are shown in **Table 2**. Recent field studies suggest that vertical transport can be accompanied by horizontal transport, leading to complex three-dimensional redistribution of transport that depends on crop cover, soil structure, and landscape features (Rillig et al., 2017b; Wan et al., 2019; Severe et al., 2025).

**Table 2:** Transport and retention pathways of microplastics in agricultural soils.

Transport or retention process	Main direction	Mechanism	Important controlling factors	Interpretation and evidence limitations	Representative references
Matrix flow and vertical leaching	Downward	Movement of suspended particles with infiltrating water through the soil matrix	Particle size, density, aggregation, pore size, infiltration rate and soil moisture	Smaller particles generally show greater mobility, but retention may increase through aggregation or attachment to soil surfaces	(Huerta Lwanga et al., 2016; Sintim and Flury, 2017; Rillig et al., 2019; Ziajahromi et al., 2026).
Preferential flow	Downward	Movement through cracks, macropores, root channels and fauna-created burrows	Soil structure, wetting–drying cycles, root architecture, shrinkage cracks and biological channels	Preferential pathways may permit transport of particles larger than the average soil pore size	(Huerta Lwanga et al., 2016; Zhang and Liu, 2018; Li et al., 2020a; Schefer et al., 2025).
Surface runoff	Horizontal	Detachment and downslope movement with overland water flow	Rainfall intensity, slope, surface cover, soil roughness and particle density	Primarily redistributes surface particles within or between fields	(Rillig et al., 2017b; Wan et al., 2019; Li et al., 2020a; Han et al., 2022; Severe et al., 2025).
Wind redistribution	Horizontal and atmospheric	Resuspension and transport of light fibers and dust-like particles	Wind speed, soil dryness, vegetation cover, particle shape and settling velocity	Fibers may remain airborne longer than compact fragments because of their shape	(Dris et al., 2016; Cai et al., 2017).
Tillage incorporation	Vertical and horizontal	Mechanical mixing and redistribution within the cultivated layer	Tillage depth, frequency, implement type and initial particle location	Mainly redistributes particles within the plough layer rather than producing unidirectional transport	(Rillig et al., 2017b; Li et al., 2020a; Wang et al., 2026; Schefer et al., 2025).
Irrigation-driven movement	Mainly horizontal near the surface; potentially vertical during infiltration	Particle mobilization by irrigation water and shallow subsurface flow	Flow rate, irrigation method, soil saturation, surface slope, particle size and aggregation	Evidence for irrigation-driven lateral movement remains limited and depends on irrigation method, flow rate, surface slope, soil saturation, soil structure, and particle properties.	(Li et al., 2020a; Zhao et al., 2022; Laermanns et al., 2021).
Root-mediated retention and redistribution	Variable	Particle entrapment by root hairs and mucilage, displacement during root growth and movement through root-created channels	Root density, architecture, mucilage, particle shape, size and water uptake	Polyester fibers may attach strongly to wheat roots, whereas compact PVC fragments may move more freely through the soil profile	(Tumwet et al., 2024; Schefer et al., 2025; Ziajahromi et al., 2026).
Fauna-assisted redistribution	Vertical and horizontal	Ingestion, egestion, burrowing and casting	Faunal species, population density, feeding behavior and soil moisture	Produces heterogeneous redistribution rather than uniform movement	(Rillig et al., 2017b; Rillig and Lehmann, 2020).
Mineral-mediated retention or release	Mobility control rather than a separate direction	Electrostatic attachment, cation bridging, heteroaggregation, competition for entry sites and possible detachment after solution-chemistry changes	Clay type, mineral charge, particle surface chemistry, pH, ionic strength and dissolved organic matter	Evidence from wheat shows that clay minerals can reduce the mobility and uptake of 200-nm PS nanoplastics; this should not be generalized automatically to larger microplastics	(Yang et al., 2025).

### 3.3 Other transport pathways and combined mechanisms

Besides vertical and horizontal transport, root-mediated transport, fauna-assisted transport, and coupled transport redistribute microplastics. Particles can be trapped, held, or directed by the roots due to the density of the root system, distribution of the root hairs, and mucilage secretion (Laermanns et al., 2021; Schefer et al., 2025). Root activity may hinder downward movement and also provide preferential channels for movement into deeper soil layers. Field evidence from Mollisols indicates that crop presence can alter the morphology, abundance, and mass distribution of soil microplastics, although the mechanisms responsible for these changes remain uncertain (Wang et al., 2022). The activity of soil fauna (earthworms, nematodes, arthropods) results in non-uniform deposition patterns and in the transport of particles from vertical to lateral/horizontal directions through ingestion, burrowing, and casting (Rillig et al., 2017b; Rillig and Lehmann, 2020). Microplastics can also carry heavy metals, pesticides, or organic pollutants that can co-migrate on these biological and physicochemical pathways (Ren et al., 2021). Combined transport mechanisms illustrate the complexity of the microplastic redistribution, emphasizing that there is a high level of patchiness, dynamism, and context-dependency in the accumulation of microplastics across agricultural landscapes.

## 4 Transformation and degradation of microplastics

The persistence of microplastics does not mean that their physical and chemical properties remain unchanged in soil. Mechanical fragmentation, oxidation, hydrolysis, microbial colonization, and partial biodegradation can alter particle size, surface chemistry, mobility, sorption behavior, and interactions with organic matter and nutrients (Rehman et al., 2025). These processes may generate smaller secondary microplastics or nanoplastics without causing complete mineralization. Transformation can increase mobility by producing smaller particles, but it may also reduce mobility by promoting aggregation or biofilm formation. The outcome depends on polymer type, soil conditions, exposure duration, and the distinction between physical fragmentation and true polymer degradation (Sander, 2019; Wang et al., 2019; Yuan et al., 2020; Zhou et al., 2020; Schöpfer et al., 2022; Wang et al., 2026). Soil degradation of biodegradable polymers such as PBAT and PLA depends on polymer composition and environmental conditions and may remain incomplete (Weng et al., 2013). Microbial enzymes can contribute to polymer depolymerization and partial biodegradation, although complete mineralization is generally slow under soil conditions (Amobonye et al., 2021). Field studies of soil-biodegradable mulch films show that degradation rates depend on material properties and environmental conditions (Griffin-LaHue et al., 2022).

### 4.1 Fragmentation

The most important physical transformation process that breaks down larger plastic debris into smaller micro- and nanoplastic particles is fragmentation. Over time, fragmentation occurs due to mechanical abrasion from tillage and machinery, root growth, and environmental stresses such as freeze-thaw cycles, wetting-drying cycles, and soil compaction (Sander, 2019; Zhou et al., 2020). Fibrous plastics, e.g., polyester and polyamide threads, may fragment into very small, irregular pieces, and films can be torn into thin fragments that remain in the topsoil (Yuan et al., 2020; Schöpfer et al., 2022). Fragmentation increases the surface area-to-volume ratio, leading to greater interaction with soil particles, organic matter, and contaminants. Field studies in recent years have also shown that the size distribution of microplastic fragments can vary significantly within an individual cropping season, with smaller fragments deposited in deeper layers and thus inaccessible to roots (Wang et al., 2019, 2026).

### 4.2 Chemical aging

Chemical aging is the surface alteration of polymers caused by interactions with soil chemistry, hydrolysis, oxidation, and photodegradation. These processes modify surface functional groups, adding carbonyl, hydroxyl, and carboxyl groups, thereby making the surface more hydrophilic and reactive (Yuan et al., 2020; Convertino et al., 2024). Chemical aging may increase soil adsorption of metals, pesticides, and nutrients, thus impacting soil chemistry and nutrient uptake (Convertino et al., 2024). The rate of aging is affected by soil pH, moisture, and microbial activity. Older microplastics have been shown to have increased sorption potential for pollutants, which can act as vector effects, carrying co-contaminants from one soil layer to another and into plants (Ren et al., 2021). Weathering of plastic particles may increase surface cracking and fragmentation, thereby generating smaller and potentially more mobile particles (Zhang et al., 2024; Wang et al., 2026).

### 4.3 Biofilm formation

Microplastic surfaces can be rapidly colonized by soil microorganisms, forming a plastisphere that may alter particle aggregation, retention, surface chemistry, and interactions with soil components (Hüffer et al., 2019; Rillig et al., 2024; Liu et al., 2025). Bacterial and fungal biofilms can reduce transport and aggregate particles, and can cause adhesion to soil aggregates or to roots. Another mechanism of polymer film degradation in biofilms is enzymatic activity, particularly in biodegradable plastics (Hüffer et al., 2019; Rillig et al., 2024). Microbial community composition on microplastics differs from that in the surrounding soil, affecting nutrient cycling, enzyme activity, and rhizosphere interactions. Biofilm formation also enables the co-transport of contaminants, since microbial exudates can bind metals, organic compounds, and pesticides, forming microplastic pollutant complexes that can affect their bioavailability to wheat roots (Rillig et al., 2024).

### 4.4 Implications for wheat and soil systems

The fragmentation, chemical aging, and biofilm formation in soil are important for wheat cultivation. Smaller particles resulting from fragmentation and aging are more likely to be transported to the rhizosphere and taken up by roots (Wang et al., 2019, 2026). The sorption capacity of aged surfaces may be higher, thereby exposing them to co-contaminants, which could impact nutrient availability and lead to oxidative stress in plants (Ren et al., 2021). Biofilm microbes attached to microplastics may alter the structure of soil microbial communities and enzyme activity, thereby influencing nutrient uptake efficiency by roots and nutrient cycling rates (Hüffer et al., 2019; Rillig et al., 2024; Liu et al., 2025). Overall, these changes make the mechanisms of particle interactions with plants and soil in such systems more complex and highlight the need to study both particle and soil properties together. Changes in particle size, surface chemistry, and microbial colonization may also influence carbon, nitrogen, and phosphorus dynamics and thereby modify particle behavior and plant responses within the soil–plant system (Rehman et al., 2025).

## 5 Effects on soil microorganisms, enzymes, and nutrient cycling

Microplastics can modify both biotic and abiotic functions in agricultural soils. In agricultural soils, they affect microbial communities, enzyme activities, and nutrient dynamics, which play a critical role in maintaining wheat productivity (Liang et al., 2019; Rüthi et al., 2020; Wang et al., 2020; Gao et al., 2021; Verma et al., 2023; Han et al., 2024; Wang et al., 2024; Rohrbach et al., 2025). The effects of these are influenced by the concentration, polymer type, particle size, and surface aging, as well as soil properties like texture, organic matter, and moisture content (Gao et al., 2021; Han et al., 2024; Rohrbach et al., 2025).

### 5.1 Impact on soil microbial communities

Microplastics create new surfaces for microbial colonization, creating a plastisphere that supports bacteria, fungi, and other microbes that are different from the surrounding soil microbial community (Rüthi et al., 2020; Gao et al., 2024b). The properties of the particles affect microbial colonization: rough surfaces and hydrophobic polymers enhance the formation of microbial colonies, whereas smooth surfaces might reduce the number of organisms (Liang et al., 2019; Rüthi et al., 2020). It has been found that the plastisphere can selectively support specific bacterial species (e.g., decomposers) while inhibiting others (e.g., nitrifiers and nitrogen-fixing bacteria), thereby altering nitrogen availability in the rhizosphere (Verma et al., 2023). Evidence from common bean indicates that microplastics can alter rhizosphere bacterial communities, but these responses may be crop- and context-dependent and should not be treated as direct evidence for wheat (Meng et al., 2023).

The location of microplastics within the soil–root interface is also important. Fibers and fragments cluster around roots, forming microhabitats with different oxygen diffusion, water holding capacity, and organic matter levels. Microbial interactions, such as competition, predation, and symbiosis, that occur among microbes on these microsites can affect plant nutrient uptake and resistance to pathogen attacks (Gao et al., 2024b; Wang et al., 2025). Studies in the wheat rhizosphere demonstrate that these microplastic fibers stimulate microbial biomass but can also reduce the activity of certain enzyme functional groups involved in nitrogen cycling, thereby exerting both stimulatory and inhibitory effects on these functions, depending on context (Liang et al., 2019; Verma et al., 2023).

### 5.2 Effects on soil enzyme activities

Soil enzymes are involved in important processes of nutrient cycling, and microplastics can affect their activities by physically blocking them, adsorbing to their surfaces, or altering their activities through microorganisms (Gao et al., 2021; Wang et al., 2024; Rohrbach et al., 2025; Ruan et al., 2025). In soils that have high concentrations of microplastics, urease activity, which is important in the mineralization of nitrogen, may be reduced in the soil because of the adsorption of these enzyme molecules on the surface of the polymer molecules, thereby decreasing the availability of the enzymes for catalysis (Gao et al., 2021). Phosphatase and β-glucosidase activities could also be altered, either stimulated by a higher microbiome on microplastics or inhibited by shading or surface adsorption of the particles (Wang et al., 2024; Ruan et al., 2025).

Further, compounds leached from microplastics, especially those that are chemically coated (e.g., stabilizers or plasticizers), can affect the metabolism of microorganisms and may serve as toxicants, impacting enzyme activity (Lan et al., 2025; Rohrbach et al., 2025). Recent research indicates that the production of enzymes and the attachment of microorganisms to the surfaces of aged microplastics are not only stimulated but also shaped by surface oxidation, suggesting that surface-aged plastic can also alter soil enzymatic function (Meng et al., 2022; Lan et al., 2025).

### 5.3 Influence on nutrient cycling

Interactions among microplastics, microorganisms, and enzymes may modify the cycling of nitrogen, phosphorus, potassium, and carbon (Wang et al., 2020; Gao et al., 2021; Han et al., 2024). Microplastic surfaces may adsorb nutrients and co-contaminants, while particle-induced changes in soil porosity, moisture, aggregation, and microbial activity may indirectly alter nutrient mobility and availability. However, wheat-specific field evidence for localized nutrient immobilization remains limited, and findings from incubation experiments or other crops should not be generalized directly to field-grown wheat. The effects of microplastics on soil microorganisms and enzymes may extend beyond nutrient availability to carbon turnover and greenhouse-gas production. Changes in soil aeration, moisture, organic-carbon availability, aggregate structure, and microbial-community composition may alter CO_2_, N_2_O, and CH_4_ emissions. Reported responses vary among polymers, concentrations, soil types, incubation conditions, and exposure periods, and the mechanisms responsible for these differences remain uncertain (Khan et al., 2024). These wider biogeochemical changes may influence the long-term functioning of wheat-growing soils, although wheat-specific field evidence is still limited.

The influence of microplastics on soil biochemical processes may also depend on the presence of other agricultural contaminants. In a pot experiment with pak choi, PLA and polyethylene microplastics, applied alone or together with oxytetracycline, altered root traits, extracellular enzyme activity, and soil CO_2_ and N_2_O emissions (Khan et al., 2024). Because this study used a different crop and included both individual and combined treatments, it should be cited as evidence of context-dependent soil and co-exposure responses rather than as direct evidence for wheat. Responses from the combined treatments should not be attributed to microplastics alone.

A rice-rhizosphere study reported that combined exposure to microplastics and arsenic altered soil nutrients and microbial communities; however, these findings should not be treated as direct evidence for wheat (Dong et al., 2021). The surfaces of microplastics can adsorb co-contaminants like heavy metals or pesticides, which can lead to microplastic contaminant complexes and change the mobility and cycling of nutrients (Wang et al., 2020; Meng et al., 2022). Furthermore, soil structural changes induced by microplastics, including decreased aggregate stability and altered porosity, can affect water retention and aeration, which, in turn, can indirectly influence microbial metabolism and nutrient turnover (Han et al., 2024; Wang et al., 2025).

### 5.4 Implications for wheat systems

Combined effects on microbes, enzymes, and nutrient cycling lead to downstream impacts on wheat growth and productivity. Microplastic-induced inhibition of nitrifiers or urease activity can reduce nitrogen availability, which in turn can impair root development, reduce photosynthetic efficiency, and yield (Verma et al., 2023; Han et al., 2024). Effects of microplastics on soil microorganisms, enzyme activities, and nutrient cycling are shown in **Table 3**. Alterations in phosphorus cycling and microbial decomposition of organic matter may affect grain quality and flour composition. Additionally, microplastics can affect wheat’s resilience to stress by altering microbial networks in the wheat rhizosphere.

**Table 3:** Effects of microplastics on soil microorganisms, enzyme activities, and nutrient cycling.

Component	Observed effect	Mechanism	References
Bacteria	Shifts in diversity and abundance	Plastisphere formation, substrate adsorption, altered oxygen/moisture	(Rüthi et al., 2020; Verma et al., 2023; Gao et al., 2024b).
Fungi	Altered abundance and diversity	Fiber entanglement, biofilm interactions, nutrient redistribution	(Liang et al., 2019; Rüthi et al., 2020; Gao et al., 2024b; Wang et al., 2025).
Enzymes	Activities increased or decreased depending on polymer type, concentration, aging, and soil conditions.	Physical blockage, adsorption, microbial stimulation, and additive effects	(Gao et al., 2021; Meng et al., 2022; Wang et al., 2024; Lan et al., 2025; Rohrbach et al., 2025; Ruan et al., 2025).
Nutrients	N, P, K, C cycling altered	Adsorption/desorption, microbial modulation, co-contaminant binding	(Wang et al., 2020; Gao et al., 2021; Meng et al., 2022; Lan et al., 2025).

Overall, the effects of microplastics on soil biological functions are diverse and dependent on polymer type, particle size, surface aging, soil characteristics, and cropping systems. It is important to understand these interactions to make plans to reduce the buildup of microplastics in wheat soils without compromising soil health and productivity.

## 6 Evidence for wheat root interactions, internalization, and root-to-shoot transport

### 6.1 Root surface adhesion and entrapment

Interactions between wheat roots and plastic particles are most clearly supported for root-surface attachment and physical entrapment. Fibers and fragments may become retained among root hairs, mucilage, adhering soil particles, and microbial biofilms. This external association may change the physical conditions around roots and influence water or nutrient acquisition without requiring particle entry into plant tissues. Microscopic observation of particles near a root or within adhering rhizosphere soil should therefore not be described as evidence of internal uptake unless the particle is clearly located inside root tissue using an appropriate imaging or chemical-confirmation method. The effects of these adhesions may alter the architecture of the root system, thereby affecting growth or biomass distribution in the plant (Li et al., 2020b; Azeem et al., 2021; Luo et al., 2022). The rhizosphere environment, represented by soil moisture, organic matter, and microbial biofilms, also influences adhesion patterns, establishing adhesion hotspots across different parts of the root zone (Li et al., 2020b; Taylor et al., 2020; Zytowski et al., 2025).

### 6.2 Evidence for root internalization

Evidence for root internalization must be separated according to particle-size class. Larger microplastic fibers and fragments are generally retained on the root surface, within mucilage, or in the surrounding rhizosphere. Nanoplastics and some submicrometer particles have a greater potential to cross root barriers. Proposed entry locations include root tips, lateral-root emergence zones, epidermal discontinuities, and apoplastic spaces. Evidence for symplastic movement through living cells is less consistent and depends on particle size, surface charge, functional groups, exposure medium, root development, and analytical method (Azeem et al., 2021; Luo et al., 2022). Detection of nanoscale particles inside root tissues demonstrates that nanoplastic internalization is possible under certain controlled conditions. It does not establish that micrometer-scale particles are routinely absorbed by wheat grown in field soil. Root-surface attachment, localization in root cracks, entry into root tissues, and entry into vascular tissues should therefore be reported as different levels of evidence.

### 6.3 Evidence for root-to-shoot transport

Root-to-shoot transport has been demonstrated mainly for nanoplastics and submicrometer plastic tracers rather than for larger microplastics. Once sufficiently small particles enter root tissues and reach vascular regions, a limited fraction may move toward stems or leaves through the transpiration stream. Transport efficiency is influenced by particle size, surface properties, aggregation, root anatomy, growth medium, soil structure, and interactions with minerals and organic matter. Some experiments with labeled nanoplastics have detected particles in wheat shoots, although most recovered particles remained associated with roots (Luo et al., 2022; Ziajahromi et al., 2026). Evidence for root interaction, internalization, and translocation of plastic particles in wheat is summarized in **Table 4**. Entry into outer root tissues does not necessarily demonstrate access to vascular tissues or systemic movement. The expressions “nanoplastic uptake” and “nanoplastic translocation” should therefore be used when nanoscale particles were examined, and these findings should not be generalized to all microplastics.

**Table 4:** Evidence for root interaction, internalization, and translocation of plastic particles in wheat.

Particle characteristics	Exposure system	Main location detected	Main finding	Evidence category	Main limitation	Citation
Fluorescent PS particles, 40 nm and 1 μm	Wheat seedlings grown in agar at 0.029 g L^−1^	Root surface and root-cap region	Both particle sizes accumulated on root surfaces, but convincing internal uptake into wheat-root tissues was not demonstrated	Root-surface association; no confirmed internalization	Agar-grown young seedlings do not represent mature wheat under field-soil conditions	(Taylor et al., 2020).
PVC fragments, 125–300 μm; polyester fibers, approximately 5,000 ± 300 μm long and 21 ± 2 μm in diameter	Wheat grown for 118 days in 70-cm sandy-soil rhizotrons at 0.24% w/w	Root surfaces and different soil depths	Polyester fibers attached strongly to wheat roots, whereas PVC fragments showed greater vertical mobility through the soil profile	External root attachment and root-mediated soil redistribution	The study did not examine cellular internalization or vascular transport	(Tumwet et al., 2024).
Europium-labeled PS particles, 200 nm	Wheat grown under hydroponic and sandy-soil conditions	Mainly roots, with limited detection in shoots	Particles accumulated predominantly in roots, while only a limited proportion was transported to shoots	Quantified root accumulation and limited root-to-shoot transport of submicrometre plastic particles	Artificially labeled particles were used, and mature wheat grain was not analyzed	(Luo et al., 2022).
PS nanoparticles of different sizes and amino- or carboxyl-modified surface groups; exposure concentration of 20 mg L^−1^	Controlled wheat-root and cellular exposure	Root tissues and root cells; particles were not detected in the vascular cylinder	Smaller and positively charged particles entered root tissues more readily, but access to vascular tissues was not demonstrated	Root-tissue and cellular internalization of nanoplastics	High controlled exposure; no evidence of transport to shoots or grain	(Zhu et al., 2022).
PS particles of 0.1, 1, and 10 μm at 50 mg L^−1^	Twenty-one-day hydroponic wheat experiment, with and without ciprofloxacin	Roots and aerial tissues	Particles of 0.1 and 1 μm entered roots, while only 0.1-μm particles were reported in aerial tissues	Size-dependent uptake and translocation under hydroponic conditions	High exposure concentration, hydroponic conditions, and antibiotic co-exposure; grain was not examined	(Ma et al., 2022).
PS and PVC nanoplastics, approximately 30 nm, at 10 mg L^−1^	Controlled wheat seedling-root exposure	Root tips, root surfaces, and root cells	Passive and energy-dependent processes were reported to contribute to nanoplastic internalization	Cellular internalization mechanism for nanoplastics	Evidence concerns nanoscale particles under controlled seedling exposure; shoots and grain were not analyzed	(Zhu et al., 2024).
PS nanoplastics, 200 nm, in the presence or absence of kaolinite and montmorillonite	Wheat seedlings exposed with and without clay minerals	Roots, shoots, and areas near lateral-root cracks	Clay minerals formed heteroaggregates with nanoplastics and reduced their accumulation in roots and shoots	Mineral-mediated control of nanoplastic uptake and transport	The findings are specific to 200-nm PS particles and should not be applied directly to larger microplastics	(Yang et al., 2025).
PET fibers, 37.8–824 μm; PE fragments, 28.1–552 μm; pristine and aged PS nanoplastics, approximately 400 nm	Wheat grown in silty-loam soil under environmentally representative exposure conditions	Larger particles around roots; aged nanoplastics in roots and the lower stem	Larger microplastics were retained mainly around roots. Pristine nanoplastics were not internalized, whereas aged nanoplastics were detected in wheat roots and the stem base	Realistic-soil root association and qualitative uptake of aged nanoplastics	Nanoplastic detection was qualitative, and accumulation in wheat leaves or mature grain was not demonstrated	(Ziajahromi et al., 2026).

### 6.4 Lateral root uptake and localized penetration

Lateral-root emergence zones contain natural discontinuities that may provide possible entry points for sufficiently small particles. Nanoplastics and submicrometer particles may accumulate in or enter these regions, whereas larger fibers and fragments are more likely to remain attached to the root surface, mucilage, or surrounding soil. Localization within a root crack should not automatically be interpreted as cellular internalization or vascular movement. Particle behavior at these sites may vary with soil moisture, root developmental stage, particle aggregation, surface aging, and mineral interactions (Azeem et al., 2021; Yang et al., 2025). Studies of lateral-root entry should report the particle size, exposure medium, imaging method, depth of tissue penetration, and evidence of vascular access.

### 6.5 Implications for wheat growth and food safety

Plastic particles may affect wheat without entering plant tissues. Root-surface attachment, rhizosphere accumulation, changes in soil pore structure, microbial communities, enzyme activity, and nutrient availability may influence wheat growth through external and soil-mediated pathways. Internalization and limited root-to-shoot transport have been demonstrated mainly for nanoplastics and submicrometer particles under controlled conditions (Li et al., 2020b; Azeem et al., 2021; Luo et al., 2022). These findings should not be generalized to larger microplastics or interpreted as evidence of substantial root-to-grain transport under field conditions. Food-safety implications should therefore distinguish systemic particle movement from foliar deposition, post-harvest contamination, and the transfer of particle-associated chemicals.

## 7 Effects on wheat growth, physiology, yield, and quality

Microplastics may affect wheat through direct contact with seeds and roots and indirectly through changes in soil structure, water availability, microorganisms, enzyme activity, nutrient cycling, and associated contaminants. Reviews across crop species have identified direct, indirect, and combined-contaminant pathways that may influence germination, root growth, physiology, biomass, and yield (Liao et al., 2019; Bao et al., 2022; Gao et al., 2024a; Zhuang et al., 2024; Jin et al., 2025; Riaz et al., 2025; Sevgi et al., 2026; Vleeming et al., 2026; Iqbal et al., 2023). However, responses vary substantially among plant species and exposure systems. Wheat results should therefore be interpreted according to polymer type, particle size, shape, concentration, aging, soil characteristics, exposure period, and growth stage. Particular care is needed when responses to nanoplastics are discussed together with responses to micrometer-scale fibers or fragments. Effects of microplastics on wheat growth, physiology, yield, and grain quality are shown in **Table 5**.

**Table 5:** Effects of microplastics on wheat growth, physiology, yield, and grain quality.

Parameter	Observed effect	Mechanism	References
Germination	Delay or reduction at high concentrations	Interference with water uptake, seed microenvironment modification	(Riaz et al., 2025; Sevgi et al., 2026).
Root architecture	Reduced elongation, altered branching	Surface adhesion, oxidative stress	(Li et al., 2020b; Azeem et al., 2021; Zytowski et al., 2025).
Photosynthesis	Decreased chlorophyll content, reduced carbon assimilation	Nutrient limitation, ROS accumulation	(Liao et al., 2019; Bao et al., 2022; Jin et al., 2025).
Biomass allocation	Altered shoot-to-root ratio	Root entanglement, nutrient uptake limitation	(Liao et al., 2019; Azeem et al., 2021).
Yield components	Potential changes in tillering, spike development, grain filling, and grain weight; mature-yield evidence remains limited	Possible nutrient limitation, root stress, altered photosynthesis, and oxidative imbalance	(Liao et al., 2019).
Grain quality	Changes in protein, gluten, starch	Nutrient imbalance, co-contaminant effects	(Gao et al., 2024a; Zhuang et al., 2024).

### 7.1 Impact on germination and early seedling development

Soil contamination with microplastics may impact wheat seed germination and early root growth. It has been found that high levels of small fragments or nanoplastics can delay germination by affecting water absorption or altering the microenvironment around seeds (Riaz et al., 2025; Sevgi et al., 2026). Fibrous particles may tangle emerging radicles, causing a reduction in lateral root emergence and root elongation. Low concentrations of microplastics could, on the other hand, have no effect or even a slight positive effect on early root growth, as this may be attributed to better soil aeration or microhabitat formation, respectively (Zhuang et al., 2024; Sevgi et al., 2026).

In durum wheat seedlings, combined heat and nanoplastic exposure altered growth and physiological responses (Fontanini et al., 2025).

### 7.2 Root architecture and biomass allocation

Root growth and root morphology (root length, density, surface area, and branching) may be altered due to the interaction between microplastics and the roots. The fibrous particles attached to the surface of the root may prevent elongation and alter the lateral root growth (Li et al., 2020b; Azeem et al., 2021; Zytowski et al., 2025). The changes affect biomass allocation and often decrease shoot:root ratios. The penetration of root tissues induces mild oxidative stress, which in turn affects root cell metabolism and, consequently, nutrient uptake efficiency (Luo et al., 2022). The effects are influenced by soil texture and organic matter content: Particle mobility is generally greater in coarse-textured soils, whereas fine-textured, clay-rich soils may promote retention near the surface or within soil aggregates (Li et al., 2020b; Taylor et al., 2020).

### 7.3 Photosynthesis and physiological performance

Microplastic-induced changes in root function can affect downstream wheat photosynthesis, transpiration, and nutrient uptake. Nitrogen and other nutrient uptake by the roots may be reduced, leading to lower chlorophyll levels and lower photosynthetic efficiency (Liao et al., 2019; Vleeming et al., 2026). The exposure to microplastics may be inducing oxidative stress by increasing reactive oxygen species (ROS), leading to activation of the antioxidant defense system in leaves, as reported in studies (Bao et al., 2022; Jin et al., 2025). Such physiological stress could negatively impact carbon assimilation, biomass build-up, and plant vigor.

### 7.4 Yield components and grain production

Evidence for the effects of microplastics on mature wheat yield remains limited. Seedling and physiological studies indicate that root stress, nutrient limitation, altered photosynthesis, and oxidative imbalance could subsequently influence tillering, spike development, grain filling, and grain weight (Liao et al., 2019). However, these potential effects require confirmation in full-season soil experiments conducted at environmentally relevant concentrations. Responses are likely to depend on polymer type, particle size, shape, aging, concentration, soil properties, and environmental conditions.

### 7.5 Effects on grain quality

The indirect effects of microplastics on wheat grain quality result from their effects on nutrient uptake and plant physiology. Changes in the available nitrogen and phosphorus can affect grain protein, gluten quality, starch composition, and micronutrient content (Gao et al., 2024a; Zhuang et al., 2024). Likewise, there is a potential risk of microplastic-associated pollutants (such as plastic additives or heavy metals) being deposited in grain, raising food safety concerns (Li et al., 2023). Current evidence indicates that systemic accumulation of plastic particles in wheat grain is limited, whereas indirect effects on nutrient acquisition and plant physiology may alter grain composition, flour performance, and end-use quality (Gao et al., 2024a; Zhuang et al., 2024).

### 7.6 Summary of mechanisms

The impact on wheat growth and yield is complex. Direct interactions (e.g., adhesion, root entanglement) and indirect effects on soil microorganisms, enzyme activity, and nutrient availability all affect plant physiological performance, biomass allocation, yield components, and grain quality. The direction and size of these effects depend on the particle size, the type of polymer, its aging process, and its concentration, as well as on the soil texture and moisture (Liao et al., 2019; Bao et al., 2022; Gao et al., 2024a; Zhuang et al., 2024; Jin et al., 2025; Riaz et al., 2025; Sevgi et al., 2026; Vleeming et al., 2026).

## 8 Potential microplastic contamination of wheat grain and food-safety considerations

Potential contamination of wheat grain may arise through systemic particle movement during plant growth, deposition on aboveground plant surfaces, and contamination introduced during harvesting, storage, milling, packaging, or laboratory analysis (Luo et al., 2022; Li et al., 2023). Current evidence for substantial root-to-grain transport remains limited and is derived mainly from nanoplastic or submicrometer-particle experiments. The relative importance of these pathways is summarized in **Table 6**. Atmospheric or irrigation-related deposition may attach fibers or fragments to leaves and spikes, but surface attachment should be distinguished from internal incorporation into developing grain (Liu et al., 2020; Li et al., 2023).

**Table 6:** Potential pathways of wheat-grain contamination and strength of available evidence.

Proposed pathway or outcome	Particle class and study system	Evidence reported	Evidence strength	What can reasonably be concluded	Main uncertainty	Representative reference
Field detection of microplastics in wheat grain	PET, PE, PVC and PS measured in grain from two wheat varieties under conventional farming	PET was reported at 5.70 ± 0.41 mg kg^−1^ and PS at 0.82 ± 0.12 mg kg^−1^; PET and PE of approximately 20–40 μm showed greater grain accumulation than PVC of approximately 70–80 μm	Emerging direct field evidence	A recent field study reports measurable polymer mass in wheat grain and indicates that particle size and shape may influence accumulation	The full methods, contamination controls, particle localization within grain and separation of uptake from surface or processing contamination must be critically evaluated	(Liu et al., 2026).
Root-to-shoot movement of submicrometre particles	Europium-labeled 200-nm PS particles in wheat grown hydroponically and in sandy soil	Particles accumulated mainly in roots; transport to shoots was limited	Moderate controlled-experiment evidence for submicrometre particles	Sufficiently small particles may move from roots to shoots under controlled conditions	No mature grain was analyzed; artificial tracer and controlled exposure	(Luo et al., 2022).
Size-dependent root and aerial-tissue transport	PS particles of 0.1, 1 and 10 μm in hydroponically grown wheat	The 0.1- and 1-μm particles entered roots; only the 0.1-μm particles reached aerial tissues	Controlled evidence, strongest for the smallest particle	Transport potential decreases strongly with increasing particle size in this experimental system	High exposure, hydroponics and ciprofloxacin co-exposure; grain not examined	(Ma et al., 2022).
Uptake under realistic soil exposure	Aged and pristine approximately 400-nm PS nanoplastics in wheat grown in silty-loam soil	Aged particles were detected in roots and lower stem; pristine particles were not detected inside wheat tissues	Limited but environmentally relevant nanoplastic evidence	Surface aging may strongly influence whether nanoplastics enter wheat tissues	Qualitative detection; no mature grain analysis; applies to nanoplastics, not larger MPs	(Ziajahromi et al., 2026).
Foliar internalization	Fluorescent PS nanoplastics applied to wheat leaves	Nanoplastics were observed in wheat leaf tissues after foliar exposure; downward movement in wheat was minimal or undetectable	Qualitative evidence for foliar uptake	Foliar exposure can produce particle internalization in wheat leaves	Nominal particle details and quantitative uptake should be taken from the full methods; mature grain was not examined	(Vleeming et al., 2026).
Foliar or atmospheric surface deposition on spikes and grain	Fibers and fragments transported by wind, rainfall or irrigation splash	Particle attachment to aboveground surfaces is a plausible external contamination route	Plausible pathway; direct wheat-grain evidence remains limited	External deposition should be separated from systemic root-to-grain transfer	Surface attachment may be mistaken for internal grain contamination unless tissues are carefully cleaned and analyzed separately	(Liu et al., 2020); Li et al., 2023).
Post-harvest contamination	Fibers and fragments introduced during harvesting, threshing, transport, storage, milling or packaging	Plastic particles may be introduced after the crop is removed from the field	Plausible and methodologically important pathway	Detection in flour or processed wheat products does not by itself demonstrate soil-to-grain transfer	Source attribution and contamination during sampling and laboratory preparation	(Aysha et al., 2024).
Indirect grain-quality effects	Microplastic or nanoplastic exposure affecting soil processes and wheat physiology	Changes in nutrient uptake, oxidative balance and plant metabolism may alter protein, starch, gluten or micronutrient composition	Some wheat-specific physiological evidence	Grain quality may change without particles entering the grain	Field relevance, exposure realism and separation of direct from indirect effects	(Gao et al., 2024a; Zhuang et al., 2024).
Transfer of particle-associated chemicals	Metals, pesticides, antibiotics and plastic additives associated with particles	Co-contaminants may desorb, become bioavailable and enter plants independently of the plastic particle	Mechanistically plausible but compound-specific	Chemical transfer must be assessed separately from plastic-particle uptake	Requires evidence of contaminant loading, release, bioavailability and grain accumulation	(Gan et al., 2025).

### 8.1 Potential pathways of grain contamination

Potential plastic contamination of wheat grain may arise through three different pathways: systemic movement during plant growth, deposition on aboveground plant surfaces, and contamination during harvesting, storage, milling, packaging, or laboratory handling. Of these pathways, root-to-grain transport is currently the least clearly demonstrated. Studies showing movement within crop plants have predominantly used nanoplastics or submicrometer tracers under controlled conditions (Luo et al., 2022). Detection in roots or shoots does not by itself demonstrate movement into mature grain. Atmospheric or irrigation-related deposition may attach fibers or fragments to leaves and spikes, but surface attachment should be distinguished from internal incorporation into developing grain (Li et al., 2023). Post-harvest contamination must also be evaluated separately because particles introduced during threshing, transport, storage, milling, packaging, or sample preparation cannot be attributed to soil-to-plant transfer. Reliable source attribution requires procedural blanks, polymer confirmation, contamination-controlled sampling, and separate analysis of grain surfaces and internal tissues.

A recent field-based study reported polymer-specific microplastic measurements in wheat grain, although independent confirmation and rigorous separation of systemic uptake, surface deposition, and post-harvest contamination remain necessary (Liu et al., 2026).

### 8.2 Factors affecting grain contamination

Particle size is an important determinant of plant interaction, but the available literature does not support a universal size threshold below which plastic particles will necessarily enter wheat grain. Evidence for internalization and vascular movement is strongest for nanoscale and some submicrometer particles, whereas larger fibers and fragments are generally retained in soil, the rhizosphere, or on plant surfaces (Luo et al., 2022). Polymer type, shape, surface charge, aging, aggregation, biofilm formation, soil texture, mineral composition, moisture, organic matter, and root development may further influence particle availability (Taylor et al., 2020; Li et al., 2023). The exposure medium is also important because particle mobility and root contact are often greater in hydroponic experiments than in structurally complex soil. Grain-contamination studies should therefore report the actual particle size and classification and should distinguish internal detection from external attachment.

### 8.3 Potential effects on grain quality and food safety

Food-safety assessment should distinguish among hazard, exposure, and risk. Microplastic or nanoplastic exposure may alter wheat physiology, nutrient acquisition, oxidative responses, and rhizosphere processes and thereby produce indirect changes in grain composition. Such changes may be relevant to crop performance and nutritional quality even when particles are not detected inside the grain (Gao et al., 2024a; Zhuang et al., 2024). Particle-associated metals, pesticides, antibiotics, and plastic additives represent possible hazards, but their importance in wheat should be assessed using evidence of release, bioavailability, plant uptake, and grain transfer (Li et al., 2023; Aysha et al., 2024). Evidence from a freshwater food-chain study shows that metal-doped nanoplastics can transfer associated metals, although the direct relevance of this finding to soil-grown wheat remains uncertain (Abdolahpur Monikh et al., 2022). Current evidence supports continued monitoring and method development but does not permit firm conclusions about the magnitude of human-health risk from soil-derived microplastic accumulation in wheat grain (Aysha et al., 2024; Gao et al., 2024a).

## 9 Straw incorporation and potential carryover to subsequent crops

Straw incorporation can modify the fate of microplastics already present in agricultural soil by changing organic-carbon availability, soil moisture, aggregation, porosity, and microbial activity. Experiments combining straw residues and microplastics have shown that residue addition can modify soil properties and ecological functions, although the direction and magnitude of these responses depend on polymer type, particle concentration, residue characteristics, and soil conditions (Chen et al., 2020; Shah et al., 2024; Yan et al., 2024). When crop residues carry externally attached plastic particles or adhering contaminated soil, their return to the field may also contribute to particle redistribution within the cultivated layer. However, evidence that wheat straw contains substantial quantities of internally accumulated microplastics remains limited, and this pathway should not be assumed without contamination-controlled measurements.

### 9.1 Effects of straw decomposition on microplastic behavior

Straw decomposition alters soil organic-matter availability, moisture, pH, aggregation, and microbial activity. These changes may influence microplastic retention, aggregation, surface aging, fragmentation, and mobility. The effects of microplastic fibers on soil aggregation and enzyme activity have been shown to depend on the amount and nature of soil organic matter, indicating that residue inputs may modify microplastic–soil interactions without necessarily transporting particles through plant tissues (Liang et al., 2021).

Microorganisms may colonize plastic surfaces and form biofilms during residue decomposition. These biofilms can modify particle attachment, aggregation, and interactions with associated contaminants. Studies combining straw and microplastic treatments have also reported changes in soil enzyme activity, microbial-community structure, and greenhouse-gas emissions (Chen et al., 2020; Shah et al., 2024; Yan et al., 2024). Nevertheless, these findings demonstrate interactions between straw decomposition and microplastics already present in soil; they do not establish that plastic particles were internally transported through wheat straw.

### 9.2 Potential effects on subsequent wheat crops

Changes in aggregation, porosity, water retention, nutrient availability, and microbial activity following straw incorporation may affect the establishment and growth of subsequent wheat crops. Microplastics remaining in soil may interact with these residue-induced changes and modify root exposure, nutrient acquisition, or microbial processes. However, the available evidence does not yet demonstrate that straw incorporation consistently increases or decreases microplastic exposure in the next wheat crop.

The effects are likely to depend on particle size, shape, polymer type, aging, soil texture, moisture, residue-management practices, and the duration of exposure. Therefore, responses observed in short-term incubation or pot experiments should not be generalized directly to multi-season wheat production.

### 9.3 Implications for long-term soil management

Repeated straw incorporation may alter the retention and redistribution of microplastics already present in the cultivated soil layer. General evidence on microplastic transport indicates that particle movement and persistence are controlled by interactions among soil structure, water flow, aggregation, biological activity, and agricultural management (Li et al., 2025). Multi-season field studies are therefore needed to determine whether residue return materially changes microplastic persistence, vertical or horizontal redistribution, rhizosphere exposure, and effects on subsequent wheat crops.

Future studies should distinguish among particles already present in soil, particles externally attached to crop residues, and particles detected within plant tissues. Contamination-controlled sampling and polymer-specific identification will be necessary before wheat straw can be considered a confirmed pathway for microplastic transfer between cropping seasons.

## 10 Conceptual framework linking microplastic fate with wheat responses

**Figure 3:**
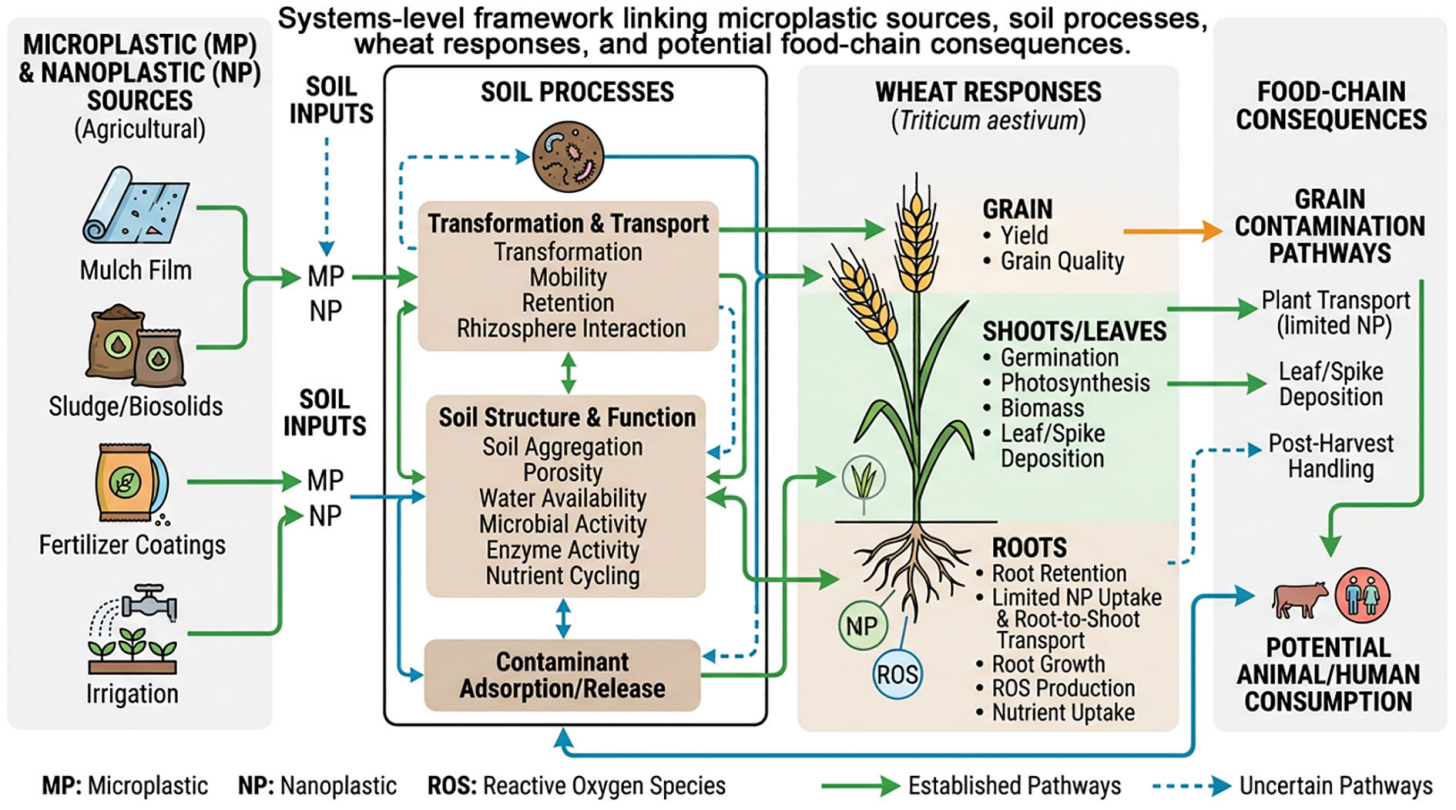
Systems-level framework linking agricultural microplastic sources, soil processes, wheat responses, and potential food-chain consequences. Agricultural sources introduce microplastics and nanoplastics into soil, where transformation, transport, retention, rhizosphere interactions, changes in soil structure and function, and contaminant adsorption or release determine plant exposure. These soil processes may affect root growth, nutrient acquisition, oxidative responses, photosynthesis, biomass production, yield formation, and grain quality. Potential grain contamination should distinguish limited systemic transport of sufficiently small particles from deposition on leaves or spikes and contamination introduced during harvesting, storage, milling, packaging, or laboratory handling. Solid green arrows indicate comparatively well-supported relationships, whereas dashed blue arrows represent uncertain or context-dependent pathways. MP, microplastic; NP, nanoplastic; ROS, reactive oxygen species.

A systems-level framework linking agricultural microplastic sources, soil transport and transformation, rhizosphere processes, wheat responses, and potential food-chain consequences is presented in **Figure 3**.

### 10.1 Entry, retention, and redistribution

The framework begins with the agricultural sources that introduce plastic particles into soil, including mulch films, sewage sludge, compost, irrigation water, coated fertilizers, atmospheric deposition, and crop residues carrying externally attached particles or adhering contaminated soil. These inputs determine the initial mixture of particle sizes, shapes, polymers, densities, surface properties, additives, and aging states. Soil texture, mineral composition, organic matter, aggregation, pH, moisture, and pore structure then influence whether particles remain near the soil surface, accumulate in the plow layer, move vertically, undergo lateral redistribution, or become concentrated in the rhizosphere.

### 10.2 Rhizosphere exposure and wheat responses

Hydrological movement, tillage, root growth, soil fauna, fragmentation, chemical aging, aggregation, and biofilm formation may alter particle mobility and biological availability. Rhizosphere exposure can affect wheat through two different routes. The first is an external or soil-mediated route involving root-surface attachment, changes in soil structure, altered microbial communities, modified enzyme activity, nutrient imbalance, and the movement of associated contaminants. The second is a possible internal route involving entry through root tips, lateral-root cracks, or other discontinuities and, for sufficiently small particles, limited vascular movement. Present evidence indicates that internalization is more plausible for nanoplastics and submicrometer particles than for larger fibers or fragments.

### 10.3 Agronomic and food-chain outcomes

Possible agronomic outcomes include changes in root architecture, water and nutrient acquisition, oxidative balance, photosynthesis, biomass allocation, yield formation, and grain composition. Food-chain outcomes must be evaluated through separate pathways: systemic transfer to edible tissues, deposition on aboveground surfaces, indirect changes in grain quality, and contamination introduced after harvest. The strength of each proposed connection depends on particle-size class, environmental realism, concentration, study duration, growth medium, analytical specificity, and contamination control. The framework should therefore distinguish well-supported soil and rhizosphere effects from pathways supported mainly by nanoplastic experiments or still uncertain under field conditions.

## 11 Conclusion

The fate and biological effects of plastic particles in soil–wheat systems are controlled by interactions among particle size, polymer type, shape, aging, concentration, soil properties, water movement, mineral composition, root activity, microorganisms, and agricultural management. These interactions influence particle retention, redistribution, transformation, and rhizosphere exposure. They may affect wheat through changes in soil structure, microbial activity, enzyme function, nutrient availability, root development, oxidative balance, photosynthesis, biomass, and yield. However, the direction and magnitude of reported effects are inconsistent. Many experiments use pristine particles, short exposure periods, single polymers, or concentrations that are difficult to relate to field conditions.

The strength of the available evidence also differs between microplastics and nanoplastics. Root-surface attachment and rhizosphere interactions are comparatively well supported for fibers and fragments. In contrast, evidence for internalization and vascular movement is derived mainly from nanoplastics and submicrometer particles studied under controlled conditions. These findings should not be applied directly to larger microplastics or interpreted as proof of substantial accumulation in mature wheat grain. Soil-derived grain contamination remains a possible exposure route, but its importance relative to foliar deposition and post-harvest contamination cannot currently be determined. Food-safety concerns should therefore be discussed as an area requiring focused investigation rather than as an established risk of substantial grain accumulation.

The most urgent research needs are standardized and contamination-controlled methods for detecting plastic particles in wheat tissues and grain; field measurements of particle abundance, size, polymer composition, and aging state in wheat-growing soils; direct comparison of field concentrations with laboratory exposure levels; clear separation of microplastic, nanoplastic, and co-contaminant effects; and multi-season studies that follow particles through soil, roots, shoots, grain, straw, and subsequent crops. Reliable exposure estimates and risk thresholds cannot be established until these limitations are addressed.
